# The complete chloroplast genome of *Cerasus humilis* (Bge.) Sok

**DOI:** 10.1080/23802359.2020.1730261

**Published:** 2020-03-02

**Authors:** Ying Tian, Jing Liu, Zhe Xu

**Affiliations:** a School of Agriculture, Ningxia University, Yinchuan, Ningxia, China;; b State Key Laboratory of Seeding Bioengineering, Ningxia Forestry Institute, Yinchuan, Ningxia, China

**Keywords:** *Cerasus humilis*, chloroplast genome, Illumina sequencing, phylogenetic analysis

## Abstract

The chloroplast (cp) genome sequence of *Cerasus humilis* has been characterized from Illumina pair-end sequencing. The complete cp genome was 158,082 bp in length, containing a large single-copy region (LSC) of 86,273 bp and a small single copy region (SSC) of 19,039 bp, which were separated by a pair of 26,385 bp inverted repeat regions (IRs). The genome contained 131 genes, including 86 protein-coding genes, 37 tRNA genes, and 8 rRNA genes. The overall GC content is 36.7%, while the corresponding values of the LSC, SSC, and IR regions are 34.6, 29.5, and 42.6%, respectively. Phylogenetic reconstruction using 59 conserved coding-protein genes clustered *C*. *humilis* within Eurosids I.

*Cerasus humilis* (Bge.) Sok is a bush fruit tree that is endemic to China; its fruits are rich in calcium and are thus also known as “Calcium fruit” (Mu et al. 2015). It is mainly distributed in the northeast, northwest, north, and other northern areas of China (Song et al. 2011). *Cerasus humilis* has long existed in the wild and studies on this species were only initiated in the 1990s (Du et al. 1993). It has a strong root system and it shows strong adaptability to saline soil, harsh winter, and drought (Yin et al. 2014). Furthermore, it has strong soil erosion resistance, therefore, it can be used for the improvement of damaged soil and environmental greening (Ha et al. 2017). Based on its ecological and economic benefits, *C*. *humilis* has become an emerging multipurpose fruit trees with a broad developmental and utilization potential. However, genome information of *C*. *humilis* has been poorly studied. In this study, we reported the complete chloroplast genome of *C*. *humilis*.

Fresh leaves were sampled from *C. humilis* in the Yinchuan Botanical Garden (Yingchuan, Ningxia, China; coordinates: 38°28′N, 106°16′E). The specimens (CH3326) were deposited in the Herbarium of State Key Laboratory of Seeding Bioengineering, Ningxia Forestry Institute (Number: 2008PC0728). Genomic DNAs were extracted using a modified CTAB method (Doyle and Doyle 1987), quantified and further sequenced on the Illumina Hiseq Xten Platform (Illumina, San Diego, CA, USA). The filtered reads were assembled using the program NOVOPlasty. The assembled chloroplast genome was annotated using Plann (Huang and Cronk 2015) and the annotation was corrected using Geneious (Kearse et al. 2012). The physical map of the new chloroplast genome was generated using OGDRAW (Lohse et al. 2013). The accurate new annotated complete chloroplast genome was submitted to GenBank with accession number *MN259192*.

The *C. humilis* cp genome is 158,083 bp in length, consisting of two inverted repeat (IR) regions of 26,385 bp, a large single-copy (LSC) region of 86,273 bp, and a small single-copy (SSC) region of 19,039 bp. The cp sequence contains 131 complete genes, including 86 protein-coding genes, 37 tRNA genes, and 7 rRNA genes. Intron-exon structure analysis indicated the majority (113 genes) are genes with no introns, whereas 16 genes contain a single intron and 2 protein-coding genes harbor two introns. The overall GC-content of the whole plastome is 36.7%, while the corresponding values of the LSC, SSC, and IR regions are 34.6, 29.5, and 42.6%, respectively.

In order to further clarify the phylogenetic characteristic of *C. humilis*, plastome of 59 reported plants were obtained from NCBI. The phylogenetic analysis was carried out with the 59 reported plant chloroplast genome sequences using PhyML (Stephane et al. 2005). The phylogenetic tree shows that *C. humilis* is within Eurosids I (Figure 1) and *C. humilis* is closely related to *C. dictyoneura*. This study provides reference for classification and identification of *C. humilis* and phytocoenosium in Rosaceae.

**Figure 1.**
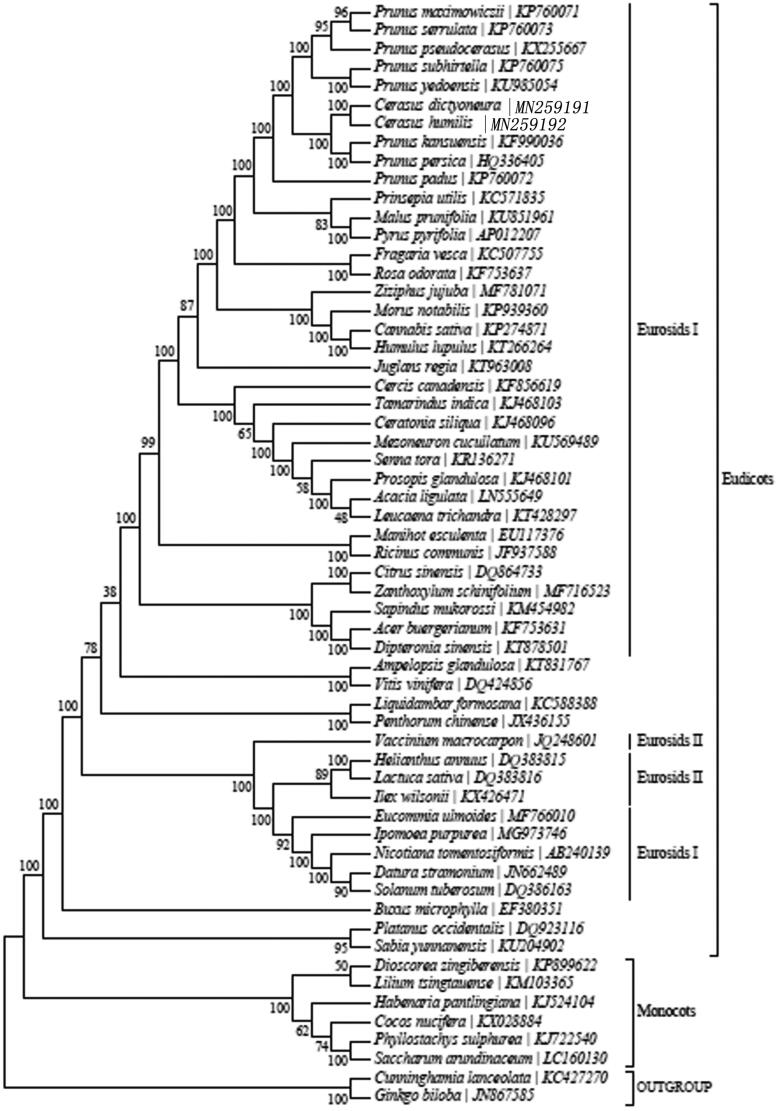
Maximum-likelihood (ML) tree of *C. humilis* and its related relatives based on the complete chloroplast (cp) genome sequences.
